# Patterns of the physical, cognitive, and mental health status of older individuals in a real-life primary care setting and differences in coping styles

**DOI:** 10.3389/fmed.2022.989814

**Published:** 2022-10-28

**Authors:** Thomas Wittlinger, Sanja Bekić, Silva Guljaš, Vlatka Periša, Mile Volarić, Ljiljana Trtica Majnarić

**Affiliations:** ^1^Department of Cardiology, Asklepios Hospital, Goslar, Germany; ^2^Faculty of Medicine, Josip Juraj Strossmayer University of Osijek, Osijek, Croatia; ^3^Department of Family Medicine, Faculty of Medicine, Josip Juraj Strossmayer University of Osijek, Osijek, Croatia

**Keywords:** complex patients, clusters, physical, cognitive and mental health status, comorbidities, copying styles, primary care setting

## Abstract

**Background:**

Physical frailty and cognitive decline are two major consequences of aging and are often in older individuals, especially in those with multimorbidity. These two disorders are known to usually coexist with each other, increasing the risk of each disorder for poor health outcomes. Mental health disorders, anxiety and depression, are common in older people with multimorbidity, in particular those with functional or sensory deficits, and frailty.

**Purpose:**

The aim of this study was to show how physical frailty, cognitive impairments and mental disorders, cluster in the real life setting of older primary care (PC) patients, and how these clusters relate to age, comorbidities, stressful events, and coping strategies. Knowing that, could improve risk stratification of older individuals and guide the action plans.

**Methods:**

Participants were older individuals (≥60, *N* = 263), attenders of PC, independent of care of others, and not suffering from dementia. For screening participants on physical frailty, cognitive impairment, and mental disorders, we used Fried‘s phenotype model, the Mini-Mental State Examination (MMSE), the Geriatric Anxiety Scale (GAS), and the Geriatric Depression Scale (GDS). For testing participants on coping styles, we used the 14-scale Brief-Coping with Problems Experienced (Brief-COPE) questionnaire. To identify clusters, we used the algorithm *fuzzy k-means*. To further describe the clusters, we examined differences in age, gender, number of chronic diseases and medications prescribed, some diagnoses of chronic diseases, the number of life events, body mass index, renal function, expressed as the glomerular filtration rate, and coping styles.

**Results:**

The most appropriate cluster solution was the one with three clusters, that were termed as: functional (FUN; *N* = 139), with predominant frailty or dysfunctional (DFUN; *N* = 81), and with predominant cognitive impairments or cognitively impaired (COG-IMP; *N* = 43). Participants in two pathologic clusters, DFUN and COG-IMP, were in average older and had more somatic diseases, compared to participants in cluster FUN. Significant differences between the clusters were found in diagnoses of osteoporosis, osteoarthritis, anxiety/depression, cerebrovascular disease, and periphery artery disease. Participants in cluster FUN expressed mostly positive reframing coping style. Participants in two pathological clusters were represented with negative coping strategies. Religion and self-blame were coping mechanisms specific only for cluster DFUN; self-distraction only for cluster COG-IMP; and these two latter clusters shared the mechanisms of behavioral disengagement and denial.

**Conclusion:**

The research approach presented in this study may help PC providers in risk stratification of older individuals and in getting insights into behavioral and coping strategies of patients with similar comorbidity patterns and functional disorders, which may guide them in preparing prevention and care plans. By providing some insights into the common mechanisms and pathways of clustering frailty, cognitive impairments and mental disorders, this research approach is useful for creating new hypotheses and in accelerating geriatric research.

## Introduction

Aging is associated with a decline in physical and mental abilities of older individuals due to intrinsic aging processes and accumulation of comorbidities (1). The decline in physical performances is best described by the concept of physical frailty, referring to the state of muscle loss and low activity, which develops progressively, as a result of the reduction in physiologic homeostatic reserves (2). In this regard, frailty is considered as a perfect risk stratification paradigm (3). The model of physical frailty phenotype was operationalized by Fried and coll. and validated for several major negative outcomes (4). The prevalence of frailty was found to increase with age, comorbidity level, some specific comorbidity patterns, and is higher in women than in men, and inherent to low socioeconomic and education background, and thus may vary between countries and population settings (5, 6). The pooled prevalence of physical frailty in EU countries, in community studies, was found to be 12% (95% CI 10–14%) (7). As we have reported before, in this sample of older (≥60 years) individuals, we found the prevalence of frailty of 14% (8).

As people age, cognitive function is expected to slightly decrease. Cognitive impairment is considered when one's cognitive performances are lower than expected for age and educational level (9). The concept of mild cognitive impairment (MCI) has been introduced in research and clinical practice to indicate the stage of cognitive decline that can be objectively measured by psychological tests, thus allowing prediction of dementia and other negative health outcomes (9, 10). The prospective epidemiologic studies indicated that physical frailty and cognitive impairment may coexist and mutually interact, potentiating the development of each other and the risk of each condition for negative health outcomes (11–14). Yet, our knowledge on the exact clustering patterns of these two conditions in an older population, and of their dynamics of change, is lacking. This is mostly due to the fact that these conditions have been studied separately, as independent entities. The cognitive frailty phenotype has been operationalized by the expert consensus panel as the coexistence of pre-frailty or frailty with MCI, to indicate the development of neuropathology changes in the context of comorbidities, and to facilitate research on the shared pathophysiology pathways for this exceptionally vulnerable patient subgroup (15, 16).

Mental disorders, anxiety and depression, are common in older people with comorbidities, in particular those with functional or sensory deficits, and with frailty, and are associated with reduced quality of life and faster functional and cognitive decline (17–20). On the contrary, positive moods and emotional wellbeing are associated with decreased risk of frailty (21). It remains to see whether anxiety/depression is on the same pathways by which some specific comorbidity patterns, and also frailty, do occur, concerning a complex interaction among intrinsic psychological vulnerability, cognitive structures and coping skills that are developed during the lifetime, stressful events, and age-associated and disease-associated neurobiological changes (20–23). Distinctly from young adults, mental disorders in older individuals are less likely to manifest with affective symptoms, and more likely with somatic symptoms, cognitive changes, and loss of interest (24, 25). This makes that these disorders in older individuals often remain unrecognized by general practitioners (GPs), which implies the need for their systematic assessment (17).

The concept of resilient aging has recently emerged, as an upgrade to the healthy aging model, promoting an idea that despite limitations and some forms of adversities, which are necessary to face when people age, it is possible to maintain a sufficient level of performance, and this may be more relevant in older age than being free from comorbidities (26). High psychological resilience is defined as one‘s capacity to bounce back from adversity, thus avoiding the pathophysiology reactions to stress, and is therefore associated with reduced risk of chronic diseases, maintaining of good mental health and physical functioning, and better survival (27, 28). The processes that underlie high psychological resilience include positive cognitive and emotional appraisal of the stressful situation, and the choice of health-enhancing and risk-diminishing behaviors. In this context, coping strategies are the key mediating resilience factors (29, 30). Coping strategies are defined as emotional, cognitive, and behavioral patterns, by which one is likely to response to stress, where positive coping promotes internal wellbeing, while negative coping promotes feelings of distress, negatively influencing an individual‘s health (31). They are categorized as problem-focused or emotion-focused, and as (pro)active or passive ones. The problem-coping strategies are based on the cognitive appraisal of the source of distress, and on taking actions to solve the problem or to prevent its negative outcomes. This strategy applies when an individual has a control over the outcome. Emotion-focused coping involves expression or regulation of emotions to lessen the feelings of distress. This strategy applies when an individual has lost the control over the situation.

There is a two-way relationship between comorbidities (functional impairments) and psychological resilience, with coping mechanisms making a link (28). In this regard, it is known that having a chronic disease or a functional disability is stressful, and requires from an individual the huge efforts to adapt to discomfort, limitations, and emotional distress, that come with these conditions (32). Coping mechanisms have been investigated in many age-related diseases, like type 2 diabetes or cancer, but taken as a single diagnosis, and more recently, in frail patients (33–35). The common conclusions of these studies are that insufficient cognitive representation of a disease or poor emotional control are associated with less pro-active care strategies and self-management behaviors, lower quality of life, and worse outcomes. It has been also observed that individuals with the same diagnosis use different coping mechanisms, and that the same coping styles are associated with similar outcomes. Only prospective study design would be able to reveal the temporal characteristics of the associations between the level of psychological resilience, emotional wellbeing, coping, and the health status. By adopting the resilience model of aging in geriatric research and medical practice, it is believed that it will provide the operative framework within which it would be possible to achieve solutions for happy and meaningful life in the face of increasing adversity (27, 36). This theory states that it is possible to achieve greater psychological resilience and emotional wellbeing of older individuals by interventions aimed at increasing social and environmental resources, which can promote their self-efficacy and change their subjective experience of living with limitations. Another way is through educational programs aimed at fostering coping abilities and self-management skills (29, 36). Implementation of the resilience theory in geriatric research, however, requires the use of more integrated measures of the aging process, to indicate functional abilities of older individuals, and more comprehensive methods of research, to represent the complex relationships among involving factors (37, 38).

Our team was among the first authors who used the benefit of new methodology approaches such as the clustering techniques of machine learning methods, to provide an integrated view on associations between physical frailty and cognitive impairments, as critical intermediates of the aging process and risk stratification tools (8, 39, 40). In this study, the aim was to identify clusters of older primary care (PC) patients with particular aggregations of physical, cognitive, and mental dysfunctions. The second aim was to describe different coping styles among the identified patient clusters. The results are expected to help GPs in risk stratification of older PC patients and in planning interventions to improve physical and mental abilities of these patients.

## Methods

### Study design and participants

It was a cross-sectional design, with the data used from general practice electronic health records (GP eHRs) and the patient interviews. Participants were older individuals (≥60), attenders of a GP setting, recruited at their regular appointments. Patients were enrolled at their first visit during a period of follow-up. Only patients who were able to visit their doctor personally, and not those dependent on the care of others, were included. The exclusion criteria included acute medical conditions, acute exacerbations of chronic conditions, current treatment with chemotherapeutic or biological agents, as well as the diagnoses of dementia and psychotic disorders. The study was conducted in the academic general medicine practice, and a skilled and knowledgeable GP collected the data. This GP has provided care for the local population for a long time and performed medical records, which ensured detailed knowledge about patients and uniformity of diagnostic criteria used in the study. To better detail the cognitive status of the patients, the GP performed interviews with their family members or caregivers by telephone calls or when they visited the doctor or during the home visits performed by the GP.

### Ethical statement

The study complied with the World Medical Association Declaration of Helsinki 2013. It was approved by the Ethics Committee of the Faculty of Medicine, the Josip Juraj Strossmayer University of Osijek (No. 641-01/18-01/01). All participants gave their written informed consent.

### Data collection procedure

The data was collected in the year 2018, in a large GP practice, in the town of Osijek, in eastern Croatia. The data collection procedure has lasted about 6 months, that is, until the sample size has reached 250 participants, that is considered a large enough sample for performing the clustering models (41). The final number of participants included in the study mounted 263. For the purpose of this study, participants were determined by many features (Table 1). The data indicating the total number of chronic diseases, diagnoses of some common chronic diseases and the total number of prescribed medications, were extracted from GPeHRs. Information on renal function (expressed as glomerular filtration rate; GFR) was derived from the chronic disease surveillance programs and was not older than a year. Information on experienced falls was taken from the eHRs and by participant interviews. Anthropometric measurements for calculating body mass index (BMI) were obtained from participants at their appointments, to detail information on their nutritional and health status.

**Table 1:** Variable description.

	**M (SD)**	**Range**	***n*** **(%)**
Age (years)	71.20 (6.43)	60.0–90.0	
Gender (M,F)			92 (35), 171(65)
Smoker			No 160 (60.8%) Ex 86 (32.7%) Yes 17 (6.46%)
Alcohol consumption			No 188 (71.5%) Infrequent 67 (25.5%) Frequent 8 (3.04%)
BMI (kg/m^2^)	30.19 (4.69)	14.30–47.05	
The number of diagnoses	3.11 (1.79)	0.0–10.0	
The number of prescribed medications	3.65 (2.15)	0.0–15.0	
The number of life events in last 3 years^*^	1.42 (1.25)	0.0–6.0	
Hypertension			• 209 (79.5%) 87 (33.1%) <10 years; 119 (45.2%) > 10 years
Type 2 diabetes			• 58 (22.1%) 25 (9.51%) <5 years; 33 (12.5%) > 5 years
Chronic heart disease			• 15 (5.70%) 14 (5.32%) NYHA lower (1–2); 1 (0.38%) NYHA higher (3–4)
Coronary artery disease			31 (11.8%)
Cerebrovascular disease			17 (6.46%)
Periphery artery disease			9 (3.42%)
Glomerular filtration rate (mL/min/1.73 m^2^)	86.85 (27.45)	18.0–191.0	
Osteoporosis			25 (9.51%)
Severe osteoarthritis			104 (39.5%)
Low back pain			103 (39.2%)
Anxiety/depression			121 (46.0 %)
Experienced falls			76 (28.9%)
Chronic obstructive pulmonary disease			13 (4.94%)
Malignant disease			29 (11.00%)
Significant visus loss			229 (87.10%)
Hearing difficulties			68 (25.90 %)
frailty_score	1.11 (1.29)	0.0–6.0	
MMSE_total	25.27 (3.40)	14.0–30.0	• 174 (66.2%)—normal global cognitive function 89 (33.2%)—decreased global cognitive function
GDS_dsysphoria	1.43 (1.80)	0.0–6.0	
GDS_affect	0.48 (1.03)	0.0–4.0	
GAS	4.81 (5.03)	0.0–27.0	
COPE-Self-distraction	6.23 (1.92)	2.0–8.0	
COPE-Active coping	6.92 (1.49)	2.0–8.0	
COPE-Denial	4.01 (1.84)	2.0–8.0	
COPE-Substance use	2.29 (0.90)	2.0–8.0	
COPE-Emotional support	4.73 (1.58)	2.0–8.0	
COPE-Use of informational support	5.67 (1.84)	2.0–8.0	
COPE-Behavioral disengagement	2.88 (1.59)	2.0–8.0	
COPE-Venting	4.21 (1.83)	2.0–8.0	
COPE-Positive reframing	6.75 (1.59)	2.0–8.0	
COPE-Planning	6.16 (1.73)	2.0–8.0	
COPE-Humor	5.40 (2.04)	2.0–8.0	
COPE-Acceptance	7.10 (1.22)	2.0–8.0	
COPE-Religion	5.43 (2.38)	2.0–8.0	
COPE-Self-blame	4.83 (1.93)	2.0–8.0	

* Operation, hospitalization, death in a family or of a loved one, illness of another close person, divorce, accident, financial loss, and relocation.

### Testing on physical frailty, cognitive impairment, mental disorders (anxiety and depression), and coping styles

To determine the level of physical frailty, we applied the Fried‘s phenotypic model (4). This model is based on five criteria, including weakness, slowness, low level of physical activity, shrinking (weight loss), and subjective feeling of exhaustion. Weakness was expressed as grip strength (GS) and measured in kilograms (kg) by the handgrip dynamometer (Jamar). Participants were required to perform the test three times with the dominant hand, in a standing position, and with a 5 min-rest in between. The highest value was used for analysis. The cut-off points were stratified by sex and BMI, as indicated by the SHARE frailty instrument for PC (42).

Men:

For BMI ≤ 24, GS ≤ 29 kg
For BMI > 24 and ≤ 28, GS ≤ 30 kg
For BMI > 28, GS ≤ 32 kg

Women:

For BMI ≤ 23, GS ≤ 17 kg
For BMI > 23 and ≤ 26, GS ≤ 17.3 kg
For BMI > 26 and ≤ 29, GS ≤ 18 kg
For BMI > 29, GS ≤ 21 kg

Slowness was defined as the walk speed in a 4,5-m walking test. Participants were observed when approaching to the GP‘s office, and the measurement was done (start-stop) when they crossed over the 4,5-m signed path. The time, in seconds, was recorded with a digital stopwatch, and gait speed (m/s) was calculated. Interpretation of the results has taken into account sex and height and the cut-off points were stratified by them (43).

Men:

≥ 7 s per height ≤ 173 cm
≥6 s per height > 173 cm

Women:

≥ 7 s per height ≤ 159 cm
≥6 s per height > 159 cm

Low level of physical activity was measured with the Physical Activity Scale for the Elderly (PASE) questionnaire, and the cut-off points were stratified by sex (44). It is a 12-item questionnaire which assesses the types of activities that are typical for older adults, including walking, recreational activities, exercise, housework, yard work, and caring for others, and uses frequency, duration, and intensity level of activities over the last week period. As the cut-off, we used the maximum score of 20% of those with the lowest scores, and it was <64 for men, and <52 for women. Shrinking was measured by unintentional weight loss (i.e., not due to dieting or exercise) of ≥5 kg or of at least 5% of body weight during the previous year. Exhaustion was determined on the basis of a positive answer to either of the following two self-reported questions of the Center for Epidemiologic Studies-Depression (CES-D) Scale: “How often did you feel that everything you did was an effort?” and “How often did you feel that you could not get going?” (45). If none of the criteria are positive, an individual was considered robust; 1–2 positive criteria indicated pre-frailty, and ≥3 positive criteria indicated frailty.

For testing participants on cognitive functions, we used the Mini-Mental State Examination (MMSE), a cognitive test that has been broadly validated, including for the Croatian population (9, 46). The maximum score of the test is 30, and the cut-off of 24/25 indicates decreased global cognitive function.

For screening participants on mental disorders, anxiety, and depression, we applied the Geriatric Anxiety Scale (GAS) and the Geriatric Depression Scale (GDS) (47, 48). These tests are suitable for use among older population, as based on the ability of these tests to quite well-discriminate symptoms of mental disorders from symptoms of cognitive and physical disorders. Since these tests have not been validated in the Croatian population before, we performed a linguistic validation and estimation of the internal factor structures of these tests, using the confirmatory factor analysis and several fit-of-model indices. The best-fitted model for the GAS test was the mono-dimensional 10-item model, whereas for the GDS test, it was the two-dimensional 10-item model, with two factors termed as dysphoria and the absence of positive mood. The both tests provided a good fit of data, with the Cronbach‘s alpha coefficient showing a value of 0.82 for the GAS test, and the values of 0.81 and 0.80 for the two domains of the GDS test.

For testing participants on coping styles, we used the 14-scale Brief-Coping with Problems Experienced (Brief-COPE) questionnaire (49). It is a 28-item questionnaire that uses a 4-point Likert scale to measure different coping responses. The coping responses may distinguish among 14 subscales, grouped into three main categories, named as: (1) problem-focused coping (including subscales: active coping, planning, instrumental support, and religion scales), (2) active emotional coping (including subscales: venting, positive reframing, humor, acceptance, and emotional support), and (3) avoidant emotional coping (including subscales: self-distraction, denial, behavioral disengagement, self-blame, and substance use). The Croatian version of the questionnaire has been validated previously (50).

### Statistical analyses

All statistical analyses were performed using the R-statistical package. Inspection of missing data indicated that the variable GFR, as a measure of renal function, was the only variable that includes missing data for total of 19 (7.22%) participants. Therefore, we excluded results of those participants only for analysis involving GFR. Furthermore, normality of distribution of numeric variables was assessed by inspecting indices of skewness and kurtosis and found that those thresholds were not exceeded for either of numerical variables. To identify clusters, created as combinations of physical, mental, and cognitive aspects of the health status of participants, we used the algorithm *fuzzy k-means* (51). This is a soft clustering technique, an alternative of the widely used *k-means* algorithm. In this approach, each data object is assigned to each defined cluster, with different membership degrees expressed as values ranging from 0 to 1. A membership of each patient to each cluster is partitioned, and higher values indicate that the point is closer to the center of a cluster.

**Figure 1:**
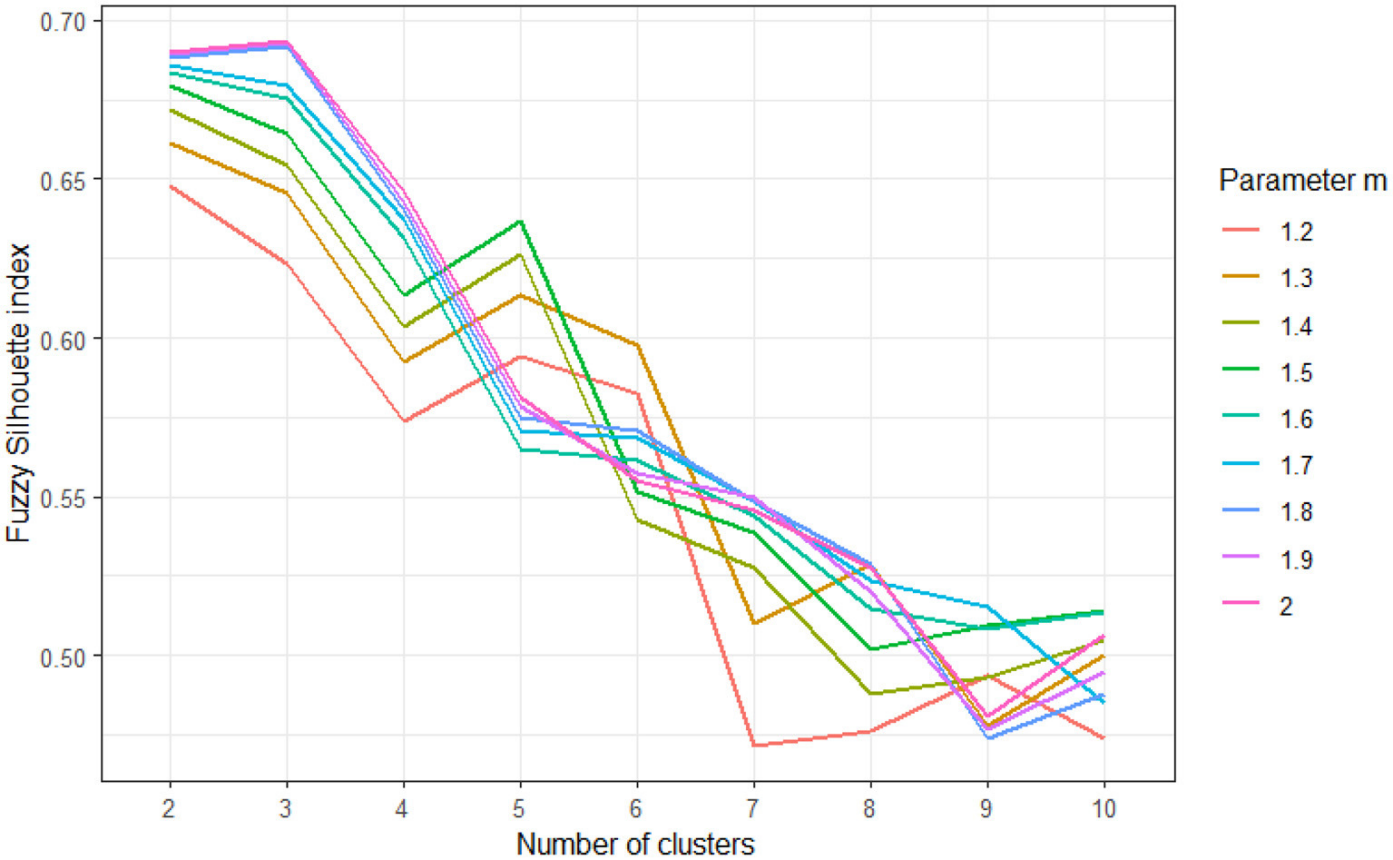
Results of fuzzy silhouette indices used to determine appropriate clustering solution, for varying number of clusters (k = 2–10) and varying values of fuzziness parameter m (m = 1.2–2). Higher fuzzy silhouette values indicate more appropriate clustering solution.

To explore the clusters, we used five measures, including the Fried‘s frailty score, three domains of newly validated GAS and GDS tests, and the MMSE test. Each of measures was standardized to *z*-scores prior to clustering, to eliminate the potential influence of different scale ranges to the final solution. We tested multiple clustering approaches, varying in number (k) of clusters to be generated, as well as the weighting exponent *m* that tunes the fuzziness of the obtained solution (51). A total of 81 different clustering solutions were evaluated by using the fuzzy silhouette index validity measure for fuzzy clustering (SIL.F) (52). Results of SIL.F of different clustering solutions are showed in Figure 1, and models with the highest SIL.F indices were evaluated for clinical significance, after which the solution with three clusters was selected for further validation.

The first cluster (*n* = 139) was the largest and included patients with MMSE values above the average, and values of measures of frailty, anxiety, and depression slightly below the average, which we called functional (FUN). The second cluster (*n* = 43) included patients with MMSE values close to the average, but with high values for anxiety, depression, and frailty, which we called dysfunctional (DFUN). The third cluster (*n* = 81) included patients whose measures of anxiety, depression, and frailty did not depart largely from the average, but whose MMSE was below the average, which we called cognitively impaired (COG-IMP).

To define the clinical profiles of participants in the clusters, we examined differences between three clusters in frequencies of many variables. Differences in frequencies of variables indicating gender, and diagnoses of some common chronic diseases and geriatric conditions, including diabetes type 2, cardiovascular disease, osteoporosis, osteoarthritis, chronic back pain syndrome, anxiety and depression, falls, hypertension, chronic obstructive pulmonary disease, cerebrovascular disease, chronic heart disease, coronary artery disease, periphery artery disease, malignant disease, and visual and hearing difficulties, were tested using the chi-squared test (**Table 3**). Statistically significant differences were further assessed by the *post-hoc* pairwise comparison *via* the Bonferroni method. Differences in age were tested using the one-way ANOVA. Differences between the clusters in the number of somatic diseases, important life events, drugs prescribed, BMI, GFR, frailty score, cognitive functions, and anxiety and depression, were assessed using the one-way Welch ANOVA, to account for differences in sample sizes (**Table 4**). The statistically significant differences were followed by the *post-hoc* pairwise comparison *via* the Bonferroni method.

As the next step, we tested whether patients from different clusters use different coping strategies, measured by the 14-subscale Brief-COPE questionnaire. Firstly, we used the one-way MANOVA to test all Brief-COPE subscales as different variables. Since the subscale “substance use” deviated greatly from the theoretically normal distribution and was rarely present in the sample, this subscale was excluded from further analysis. There was no presence of multi-collinearity among other Brief-COPE subscales, as indicated by the correlation matrix analysis (all *r* lower than 0.90) (53). The homogeneity of variance was tested using the Levene's test. Since the one-way MANOVA indicated that there are significant multivariate differences between the clusters, we have proceeded with univariate analysis of differences in individual coping strategies. Differences in coping strategies were explored with one-way ANOVA for subscales with equal variances and with Welch's one-way ANOVA for subscales with unequal variances (**Table 5**). We further assessed the significant findings with *post-hoc* pairwise *t*-test comparisons, using the Bonferroni‘s *p*-value adjustment for multiple comparisons and the Welch's *t*-test, when variances were unequal.

## Results

The main characteristics of participants in this sample were that they were mostly overweight or obese and burdened with multiple comorbidities, and that women were prevailing over men. The most prevalent disorders included diagnoses of hypertension, anxious-depressive disorders, osteoarthritis, and low back pain, and self-reported vision impairment, as well. The current smoking or alcohol consumption behaviors were rare (Table 1).

**Figure 2:**
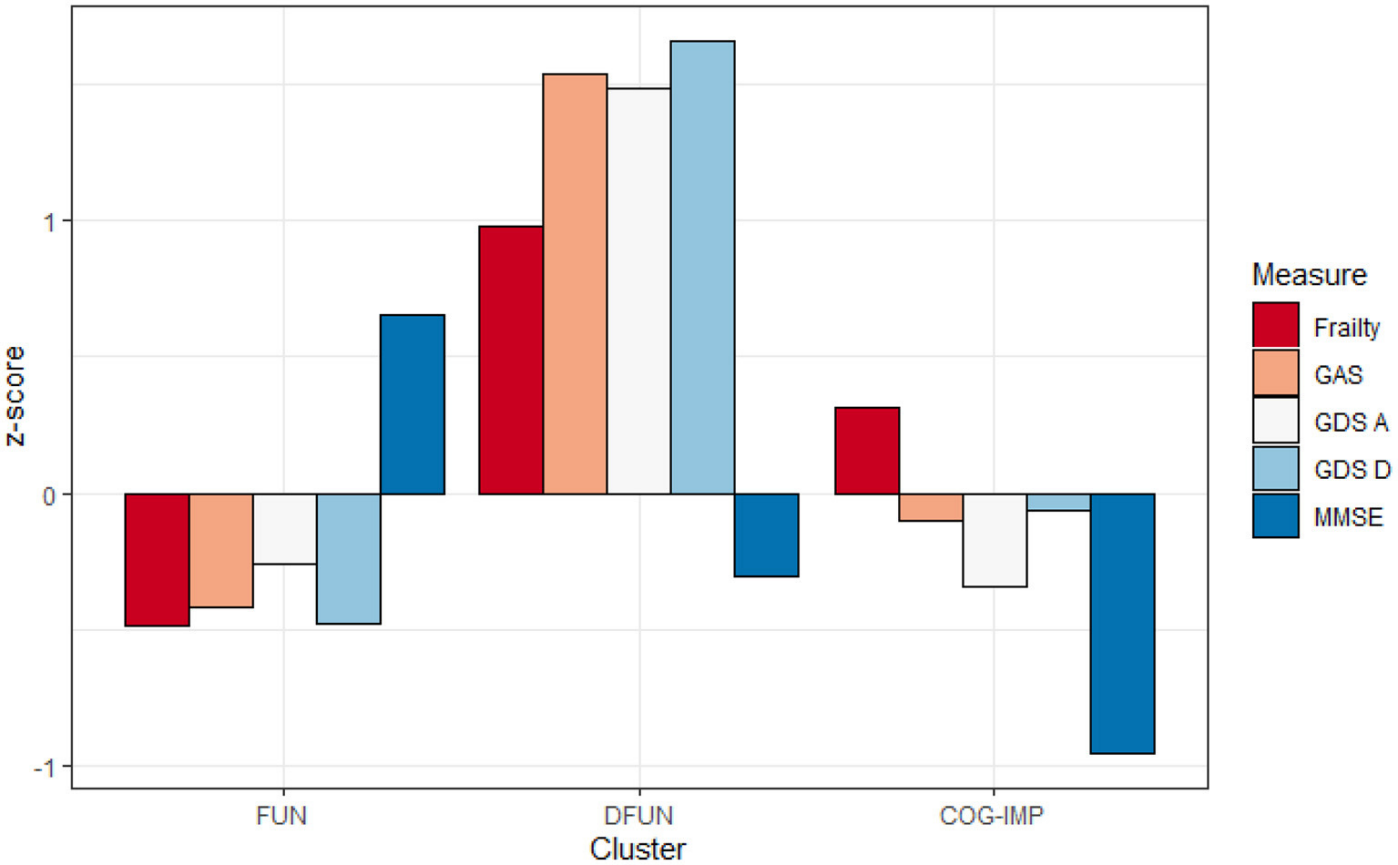
Average values of frailty score, Geriatric Anxiety Scale (GAS), Geriatric Depression Scale—lack of positive affect (GDS A), Geriatric Depression Scale—dysphoria (GDS D), and Mini-Mental State Examination (MMSE), expressed as *z*-scores in three identified clusters: Functional (FUN), Dysfunctional (DFUN), and Cognitive impaired (COG-IMP).

In the three-cluster model, participants were dispersed among three clear-cut profile models, indicating (1) those who are physically robust (cluster FUN), (2) those who show features of cognitive dysfunction (cluster COG-IMP), and (3) those who present with a combination of physical frailty and mental disorders, anxiety and/or depression (cluster DFUN; Figures 1, 2). Participants in two pathologic clusters, DFUN and COG-IMP, were in average older and had more somatic diseases, compared to participants in cluster FUN (**Table 4**).

According to the described criteria for assessing physical frailty, the prevalence of frailty was about 14%, and there were equal parts of those who were pre-frail or robust (Table 2). Regarding particular components of the physical frailty model, the most prevalence one was “low grip strength,” following by “low level activity” and “exhaustion.” The components “low level activity” and “slow walking,” were more markedly expressed in women than in men. In most of patients, global cognitive function was normal or mildly disturbed, with about a third of them having MCI (Table 3).

**Table 2:** Prevalence of frailty in sub-criteria of the Fried's frailty test.

	**Total sample**	**Men**	**Women**		
	***N*** **(%)**	***N*** **(%)**	***N*** **(%)**	**χ^2^ (df)**	***p***
Weight loss	36 (13.7%)	11 (12.1)	25 (14.5%)	0.302 (1)	0.583
Exhaustion	61 (23.2%)	17 (18.7%)	44 (25.6%)	1.59 (1)	0.207
Low level activity	68 (25.9%)	16 (17.6%)	52 (30.2%)	4.97 (1)	0.026
Low walking time	46 (17.5%)	9 (9.9%)	37 (21.5%)	5.57 (1)	0.018
Low grip strength	72 (27.4%)	26 (28.6%)	46 (26.7%)	0.100 (1)	0.752

%, within-category percentage.

**Table 3:** Differences in frequencies of gender and some diagnoses of chronic diseases and geriatric conditions between three identified clusters.

		**FUN**		**DFUN**		**COG-IMP**				
**Characteristic**		***f***	**%**	***f***	**%**	***f***	**%**	**χ^2^**	**df**	***p***
Gender (female)		85	61.2	31	72.1	56	69.1	2.460	2	0.292
Smoker	Yes	1	1.2	9	6.5	7	16.3	11.7	4	0.020
	No	55	67.9	84	60.4	21	48.8			
	Ex	25	30.9	46	33.1	15	34.9			
Alcohol	No	58	71.6	91	65.5	39	90.7	11.7	4	0.020
	Infrequent	22	27.2	42	30.2	3	7.0			
	Frequent	1	1.2	6	4.3	1	2.3			
Hypertension		68	84.0	106	76.3	35	81.4	1.97	8	0.373
Type 2 diabetes	Yes	33	23.7	7	16.3	21	25.9	1.517	2	0.468
Chronic heart disease		8	9.9	4	2.9	3	7.0	4.82	2	0.090
Coronary artery disease		8	9.9	15	10.8	8	18.6	2.34	2	0.311
Cerebrovascular disease		4	4.9	6	4.3	7	16.3	8.22	2	0.016
Periphery artery disease		3	3.7	2	1.4	4	9.3			0.033^*^
Osteoporosis	Yes	8	5.8	9	20.9	8	9.9	8.810	2	0.012
Osteoarthritis	Yes	45	32.4	22	51.2	37	45.7	6.690	2	0.035
Chronic low back pain	Yes	49	35.3	23	53.5	31	38.3	4.623	2	0.099
Anxiety/depression	Yes	52	37.4	30	69.8	39	48.1	14.058	2	<0.001
Falls	Yes	31	22.3	20	46.5	25	30.1	9.588	2	0.008
Chronic obstructive pulmonary disease		4	4.9	6	4.3	3	7.0	0.495	2	0.781
Malignant disease		12	14.8	12	8.6	5	11.6	2.01	2	0.366
Significant visus loss		70	86.4	121	87.1	38	88.4	0.095	2	0.953
Hearing difficulties		20	24.7	31	22.3	14	39.5	5.17	2	0.084

Clusters: FUN, Functional; DFUN, Dysfunctional; COG-IMP, Cognitively impaired; f, frequency; %, within cluster percentages, indicating percentage of a given category within a cluster; χ^2^, chi-square; df, degrees of freedom.

* Fisher exact test.

There were no differences in observed frequencies of males and females and in diagnoses of type 2 diabetes, chronic back pain, hypertension, chronic obstructive pulmonary disease, malignant disease, cardiovascular disease, including both chronic heart disease and coronary artery disease, and in significant visus loss and hearing difficulties, between participants in the clusters. Significant differences were found in smoking and alcohol drinking behaviors and in diagnoses of osteoporosis, osteoarthritis, anxiety/depression, cerebrovascular disease, and periphery artery disease (Table 3).

Patients from cluster COG-IMP were more often smokers but they reported more frequently that they do not drink alcohol, compared to patients from cluster FUN and DFUN (Table 3). Patients from cluster DFUN had more diagnosis of osteoporosis and experienced more falls, compared to patients from cluster FUN, while there were no significant differences in other pairs of clusters. Although the analysis of differences indicated that there are significant differences between the clusters in the diagnosis of osteoarthritis, the *post-hoc* analysis did not specifically selected any pair of clusters. Furthermore, more patients in cluster DFUN were diagnosed with anxiety/depression compared to cluster FUN. There were no significant differences between other pairs of clusters. Furthermore, patients from COG-IMP cluster had more diagnoses of cerebrovascular disease and periphery artery disease compared to participants from FUN and DFUN cluster whose prevalence of those diseases was similar (Table 4).

**Table 4:** Differences between patients in the clusters in the number of somatic diseases, important life events, the number of drugs prescribed, body mass index (BMI), glomerular filtration rate (GFR), and parameters used to identify clusters.

	**FUN**	**COG-IMP**	**DFUN**					
**Characteristic**	**M ±SD**	**M ±SD**	**M ±SD**	***F***	**DF1**	**DF2**	***P***	***p post-hoc***
Age	69.0 (5.33)	73.4 (7.05)	73.9 (6.50)	19.8	2	99.7	0.001	FUN < DFUN, COG-IMP < 0.001
The number of somatic diseases	2.71 (1.557)	3.93 (2.017)	3.35 (1.853)	8.39	2	100.55	< 0.001	FUN < DFUN < 0.05 FUN < COG-IMP < 0.01
Life events	1.34 (1.207)	1.84 (1.213)	1.33 (1.294)	3.02	2	109.32	0.053	
Drugs	3.22 (1.907)	4.79 (2.396)	3.77 (2.187)	8.15	2	101.74	< 0.001	FUN < COG-IMP < 0.001
BMI	30.34 (4.228)	29.61 (5.808)	30.25 (4.836)	0.29	2	99.57	0.750	
GFR	92.28 (25.904)	77.61 (26.258)	82.51 (28.810)	6.04	2	99.62	0.003	DFUN < FUN < 0.005 COG-IMP < FUN < 0.01
Frailty score	0.48 (0.64)	1.52 (1.23)	2.37 (1.69)	46.7	2	84.1	< 0.001	FUN < COG-IMP < DFUN < 0.001
MMSE	27.5 (1.54)	22.0 (2.59)	24.2 (3.75)	156.00	2	86.2	< 0.001	COG-IMP < DFUN < FUN < 0.001
GDS dysphoria	0.57 (0.96)	1.32 (1.50)	4.42 (1.16)	194.00	2	99.7	< 0.001	FUN < COG-IMP < DFUN < 0.001
GDS affect	0.22 (0.51)	0.12 (0.37)	2.00 (1.62)	28.2	2	94.9	< 0.001	COG-IMP, FUN < DFUN < 0.001
GAS	2.72 (2.63)	4.31 (3.35)	12.53 (6.25)	54.4	2	90.3	< 0.001	FUN < COG-IMP < DFUN < 0.001

Results in Table 4 show that patients from different clusters did not differ in the number of life events and BMI. On the contrary, there were differences in the number of somatic diseases, so that patients from cluster FUN had lower number of somatic diseases compared to patients from both, DFUN and COG-IMP clusters. Patients from cluster DFUN and cluster COG-IMP used more drugs than patients from cluster FUN. There were also differences between clusters in degrees of renal function decline, as measured by GFR. Patients from cluster DFUN and cluster COG-IMP had lower values of GFR compared to patients in cluster FUN.

As expected, variables used to select clusters, the frailty score, the MMSE score, the scores achieved on GAS test and two domains of GDS test, showed significant differences between the clusters (Table 4). Patients from FUN cluster had lower frailty score (better physical fitness) and showed lower levels of anxiety (GAS test) and dysphoria (GDS-dysphoria domain), compared to patients from DFUN and COG-IMP clusters. Patients from COG-IMP cluster had the lowest global cognitive function, as measured by the average MMSE score, and patients from DFUN cluster had the worst results on the anxiety scale (the GAS test) and the dysphoria and affect domains of the GDS test (indicating higher level of anxiety and depression).

As can be seen from Table 5, patients from different clusters differed in six (out of a total of 14) coping strategies, including “behavioral disengagement,” “denial,” “positive reframing,” “religion,” self-blame,” and “self-distraction.” The *post-hoc* analysis has shown that patients from FUN cluster used “behavioral disengagement” as a coping strategy significantly less often than patients from clusters DFUN and COG-IMP. Similarly, patients from cluster FUN were less prone to use “denial” as a coping strategy compared to patients from clusters DFUN and COG-IMP. Furthermore, patients from cluster DFUN used “positive reframing” significantly less often than patients from clusters FUN and COG-IMP. In contrast to this, patients from cluster DFUN use significantly more often “religion” than patients from clusters FUN and COG-IMP. Patients from cluster DFUN were more likely to use “self-blame” than patients from clusters FUN and COG-IMP. Finally, patients from cluster COG-IMP used “self-distraction” more often than patients from cluster FUN.

**Table 5:** Differences in coping strategies between patients in the clusters.

	**FUN**	**DFUN**	**COG-IMP**					
**Coping strategy**	**M ±SD**	**M ±SD**	**M ±SD**	***F***	**DF1**	**DF2**	***p***	***P*** **(*post-hoc*)**
Acceptance	7.122 (1.188)	6.860 (1.552)	7.173 (1.082)	0.99	2	260	0.374	_
Active coping	7.129 (1.356)	6.744 (1.544)	6.667 (1.651)	2.87	2	260	0.059	_
Behavioral dis- engagement	2.576 (1.330)	3.395 (2.002)	3.123 (1.669)	5.35	2	96.54	0.006	FUN < DFUN, COG-IMP < 0.05
Denial	3.612 (1.675)	4.837 (2.203)	4.247 (1.743)	8.74	2	260	< 0.001	FUN < DFUN < 0.001 FUN < COG-IMP < 0.05
Emotional support	4.647 (1.493)	5.116 (1.930)	4.679 (1.515)	1.08	2	102.18	0.342	_
Humor	5.626 (2.012)	4.791 (2.088)	5.346 (2.032)	2.83	2	260	0.061	_
Planning	6.345 (1.727)	6.209 (1.846)	5.827 (1.657)	2.33	2	260	0.099	_
Positive reframing	7.036 (1.293)	5.791 (2.210)	6.765 (1.469)	6.43	2	95.55	0.002	DFUN < FUN < 0.01 DFUN < COG-IMP < 0.05
Religion	5.288 (2.423)	6.302 (2.144)	5.222 (2.340)	4.06	2	114.87	0.020	DFUN > FUN, COG-IMP < 0.05
Self-blame	4.705 (1.932)	5.628 (1.813)	4.605 (1.889)	4.66	2	260	0.010	DFUN > FUN, COG-IMP < 0.05
Self-distraction	5.971 (1.989)	6.047 (2.070)	6.778 (1.605)	5.77	2	109.07	0.004	COG-IMP > FUN < 0.01
Use of informational support	5.698 (1.776)	5.512 (1.919)	5.704 (1.913)	0.19	2	260	0.829	_
Venting	4.180 (1.795)	4.465 (1.919)	4.111 (1.844)	0.55	2	206	0.575	_

## Discussion

This is the first attempt in geriatric research to cluster older individuals according to the level of physical frailty, cognitive impairment, and symptoms of mental disorders, anxiety and depression. As evidenced by the recently published papers, and by our previous work, characteristics of patients in the sample, including age and gender structures, and the prevalence of particular chronic disorders, could have influenced the cluster creation (40, 54). It has been shown e.g., that when physical frailty is fully developed, this allows for the cognitive frailty phenotype to form a cluster (8, 55). Furthermore, the severity or a duration of some disorders could be more important than just the prevalence of these disorders in influencing comorbidity profiles of patients in the clusters, as it was the case in this study with the sensory organ impairments, vision and hearing loss. These impairments did not show significant differences between the clusters, despite their high prevalence in the patient sample, and despite the fact that evidence indicated their associations with frailty (56). That shared predisposing factors or pathophysiology pathways between comorbid disorders and frailty/cognitive impairments could be of importance for their associations, the proof in this study is that the diagnoses of chronic obstructive pulmonary disease and malignant disease did not distinguish between the clusters, although evidence indicated their close associations with frailty (57). Also, illness subjective perception, and the type of coping with the disease, could have a role in frailty or cognitive impairment manifestation and cluster creation (58). That there is the need to include a wider scope of variables to describe the patient health status, and to use even more refined analytical method, that would be able to represent the complexity of the variable associations, the proof in this study is also the fact that hypertension, although the most prevalent disease in the patient sample, did not significantly differ among the clusters. However, this association could have been indirect, *via* hypertension-related comorbidities such as type 2 diabetes, cardiovascular disease and chronic kidney disease, which are known as the most important single diseases associated with frailty and cognitive impairment, and which also in this study showed differences among the clusters (59–61).

It can be seen from Figure 2 that frailty does not present alone but rather together with even small rates of cognitive impairment. The opposite is also true; when cognitive impairment is the dominant cluster feature, it is accompanied even by weak physical frailty. These findings support the theory of the coexistence of frailty and cognitive impairments, with each disorder manifesting its own rates of progression (12–14). Mild symptoms of mental disorders, demonstrated by patients in COG-IMP cluster, may be due to the overlap of these symptoms with symptoms of cognitive disorders, which are the hallmark of patients in this cluster. This assumption is supported by higher levels of anxiety and dysphoria, as prominent symptoms of mental disorders of patients in this cluster, and also evidence suggests apathy and irritability as to be the common signs of pre-dementia state (62). These results thus support the view on the need for simultaneous screening of older PC patients with multiple comorbidities on cognitive impairment and mental disorders (63). The heterogeneity of phenotypes, as the major characteristic of older individuals, especially those with multiple comorbidities, was also demonstrated by participants in this study, as supported by great variations of patients in the sample in many features, such as BMI, renal function, and the number of comorbidities and prescribed medications (64). The heterogeneity of phenotypes makes that classification and risk stratification of older individuals are difficult. Clustering based on major functional disorders, physical frailty, cognitive impairment, and mental disorders, as we have already demonstrated in our previous work, could be a promising approach to compressing this heterogeneity into a limited number of phenotypes, which are expected to have a prognostic importance (8, 39, 40, 65).

As indicated by lower number of disorders, members of cluster FUN were much healthier than those of two pathologic clusters, DFUN and COG-IMP. As the evidence also suggests for individuals following healthy aging trajectories, individuals in this cluster mostly used health-promoting coping mechanisms, such as active problem-solving strategies (of a borderline significance) and positive emotional and/or cognitive appraisal of stressful situations (indicating by positive reframing), which might have rendered them capable of diminishing the hazardous effects of emotional distress on the body and mind, and of slowing down the development of comorbidities (28). In this regard, positive reframing is mentioned in the literature as the coping mechanism which can help individuals develop high psychological resilience, by boosting the one‘s capacity for adaptation on adversities (31). The evidence emphasizes the importance of the pre-existing psychological resources for predicting future development of chronic medical conditions, with health behaviors and the current homeostatic resources (the level of comorbidity development) having the mediating role (66). There is a bi-directional interaction between the health status and coping strategies that an individual employs (32). It is not possible therefore, from this cross-sectional and pattern recognition study, to realize whether positive coping mechanisms are signs of patients' inner strength, thus being the cause of good health later in life, or rather, the positive coping style is the consequence of well-preserved health status (29, 67, 68). Nevertheless, the growing awareness on the critical role of proactive behavioral adaptations in ameliorating adverse effects of contextual stressors, poses a hope that by screening population on psychological factors at age of 50, that is, in time when chronic diseases begin to emerge at higher rates, GPs would be able to select individuals with fewer psychological resources, such as those with incoherent self-concepts or those who are lack of positive emotions and optimism, to be candidates for coping and behavioral adaptation programs (30, 69, 70).

A higher degree of comorbidity, in individuals in cluster DFUN, as evidence also suggests, is usually associated with higher prevalence of frailty and mental disorders (5, 17). The coping strategies that were found to associate with this cluster, “behavioral disengagement” and “denial,” are components of the emotion-focused strategy group. Beliefs and cognitive constructs underlying these coping strategies suggest that by avoiding some life activities it would be possible to decrease distress that arises from experience of living with unpleasant symptoms associated with chronic health conditions (71). According to this philosophy, one usually restricts his/her responsibility in caring for self and becomes more and more passive, resigned, and focused on symptoms, which can lead to the overuse of medications, deepening of somatization, and the progression of functional decline (34). These strategies have been confirmed in a number of studies and in association with different chronic diseases, notably including type 2 diabetes and musculoskeletal pain syndromes (72, 73).

As we can learn from the literature, some older individuals with cardio-metabolic conditions, such as type 2 diabetes, may use active, problem-focused coping strategies, while some others, more women, may fall into resignation, denial and isolation, and in searching for means by coping with negative emotions; this negative coping style is associated with distress, depression, somatization, and poorer outcomes (34, 72–74). The former scenario is likely to associate with individuals in cluster FUN and the latter one with individuals in cluster DFUN. This assumption is based on our results showing that the diagnoses of type 2 diabetes, coronary artery disease, and chronic heart disease, were present at similar rates in both clusters, FUN and DFUN, but the difference was in the level of comorbidity. The physical health status of individuals in cluster DFUN has deteriorated to higher degrees, as demonstrated with lower renal function, the use of more drugs, and the prominent present of musculoskeletal diseases in comorbidity profile. This higher level of comorbidity, in individuals in cluster DFUN, is also associated with disabilities such as chronic back pain and fall-related limitations in mobility, and also with frailty. The higher level of frailty in cluster DFUN, compared to cluster FUN, can be explained by the fact that chronic renal failure in older individuals develops in the presence of type 2 diabetes and cardiovascular disease, and is associated with the prominent muscle mass loss and high risk of frailty (75). In this context, difficulties which are often associated with musculoskeletal diseases, including muscle loss, mobility limitations, and chronic pain, may be even more exaggerated (74). Therefore, the emotion-focused types of coping strategies, as it is in individuals in cluster DFUN, could be a characteristic of older individuals with higher level of comorbidity, and corresponding to the presence of disabilities and frailty. As the evidence suggests, individuals in cluster DFUN, compared to those in cluster FUN, might have had fewer psychological resources and higher predisposition for anxiety and depression, which could have fueled the development of comorbidities and functional decline (28, 32, 74). In fact, it is more and more clear that the psychological adjustment to a new situation is a complex and dynamical process, driven by multiple factors, in which maladaptive coping mechanisms constantly change the sense of self, leading to the *vicious cycle* of the development of comorbidities and functional decline. In this context, it is possible to understand the results of this study showing that the mechanisms that were found to specifically distinguish individuals in cluster DFUN from those in other two clusters, were “religion” and “self-blame.” These mechanisms draw upon beliefs that illness is a punishment and unconditional faith, which lead to being passive and to one's losing the control over the situation (71). People possessing such mechanisms may be considered as stubborn and strongly resistant to change.

As the knowledge gained so far teaches us, there is no specific mechanism that would be associated with a particular chronic health condition but rather a range of mechanisms may overlap across diverse conditions (32). The same has been confirmed by the results of this study, where a set of maladaptive mechanisms was shown to characterize individuals in pathologic cluster DFUN, and there was an overlapping in a part of the spectrum between two pathologic clusters, DFUN and COG-IMP. The information burden, that is usually associated with multimorbidity and polypharmacy, could also contribute to this *vicious cycle*, by mechanisms such as low engagement of older individuals with healthcare providers‘ recommendations and low adherence to health self-management (76).

Our results indicated that individuals in two pathologic clusters, DFUN and COG-IMP, shared negative coping mechanisms, “behavioral disengagement” and “denial,” which may correspond with the fact that there were similar rates of diagnoses of type 2 diabetes and cardiovascular disease, and of renal function decline, in these two clusters. These all conditions are known to lead to the brain vascular changes which may underlie cognitive decline and dementia (77). More developed generalized atherosclerosis (as indicated with higher proportion of individuals with the diagnosis of periphery artery disease), and greater participation of the diagnosis of cerebrovascular disease, in cluster COG-IMP, may justify for higher level of cognitive decline in patients in this cluster. The average frailty level was low and at the stage of pre-frailty. This is different from cognitive frailty cluster, found in our previous work performed on the same sample, where measures of mental disorders have not been included in the process of cluster creation, indicating that information on symptoms of mental disorders may be used to better discern the co-existence between frailty and cognitive impairments (8, 40). This also implicates the need for further research that would be aimed at more precise definition of neurobiological pathways that may stay in the background of cognitive decline of older individuals, concerning the relationship between the vascular brain pathology and the direct neuronal apoptosis (of the Alzheimer's dementia type). Knowing more on associations of these pathways with clinical characteristics of patients with MCI, would help GPs selecting patients who would benefit the most from the cognitive restructuring rehabilitation programs (78).

The main feature of individuals in cluster DFUN, however, is frailty, while cognitive dysfunction is not expressed at a greater extent, which does not support the significant impact of cardio-metabolic disorders on cognitive impairments. That means, that the common burden of somatic diseases, in individuals in this cluster, has led to the stronger derangement of homeostatic mechanisms that are associated with frailty, than it is the case in cluster COG-IMP. The pieces of information which may add to clarification of these inconsistences between the level of frailty and cognitive impairment in individuals in clusters DFUN and COG-IMP are associated with the difference in the expression of musculoskeletal diseases between these two clusters. By mechanisms of sarcopenia and reduced mobility, these diseases might have contributed to higher levels of frailty in individuals in cluster DFUN (79, 80). The close association of musculoskeletal diseases with chronic pain, poor sleep quality, and anxious-depressive disorders, may also explain higher burden of individuals in cluster DFUN with symptoms of mental disorders, compared to individuals in cluster COG-IMP (73, 81). The marked expression of symptoms of mental disorders, found in patients in cluster DFUN, may also suggest psychological distraction as a mechanism of the possible importance for cognitive impairments, in patients in this cluster (82). Similar to our results, indicating a range of negative coping mechanisms that were employed by patients in cluster DFUN, other authors also found that older frail individuals with mental disorders are especially prone to passive coping mechanisms and neglecting, and that they are reluctant to help-seeking behaviors (79).

In contrast to individuals in cluster DFUN, individuals in cluster COG-IMP, whose major characteristic was cognitive decline, were less burdened with anxiety/depression. The coping mechanism found to most specifically determine individuals in this cluster was “self-distraction,” that is, a detachment from the situation, by means of being less emotional (32). It does make sense, when counting for the fact that apathy and inhibition are the hallmark neuropsychiatric symptoms in pre-dementia states (24, 62).

Finally, gender bias (in this study indicated by the predominance of women over men in the whole sample, and this proportion justified across the clusters) could have influenced the constitution of the clusters, resulting in the relative clear-cut separation of pathological clusters into one mostly represented with frailty (cluster DFUN), and another one mostly represented with cognitive impairment (cluster COG-IMP). In this regard, women are considered more prone than men to anxiety/depression, multimorbidity, musculoskeletal disease, and frailty (as represented by cluster DFUN), and also for Alzheimer's dementia (as represented by cluster COG-IMP) (4, 9, 19, 74, 80). Not only in predisposition for particular diseases, gender bias may influence differences in coping styles in individuals suffering from the same chronic disease. In this regard, in patients with type 2 diabetes, women were found to be more prone than men to negative coping styles such as resignation, protest, or isolation, while avoidance was found to associate with distress and depressive symptoms (34).

## Conclusion

For the first time in geriatric research, we identified clusters as combinations of the two main age-related functional impairments, physical frailty and cognitive decline, with the addition of measures of mental disorders, anxiety and depression, as major drivers of age-related functional decline, of how they appear in the real life setting of older PC patients. This research approach can be used to comprehend heterogeneity of older individuals but in a way to improve patient risk stratification. By relating age, gender, common comorbid disorders, and coping mechanisms, to the identified clusters, it may help getting better insights into the common mechanisms and pathways that stay behind clustering of physical frailty, cognitive impairments, and mental disorders, and into behavioral and coping patterns of patients with similar comorbidity patterns and functional disorders. The model presented in this article could help GPs to further individualize prevention and care plans.

## Data availability statement

The original contributions presented in the study are included in the article/supplementary materials, further inquiries can be directed to the corresponding author.

## Ethics statement

This study was approved by the Ethics Committee of the Faculty of Medicine, the Josip Juraj Strossmayer University of Osijek (No. 641-01/18-01/01). All participants gave their written informed consent to participate in this study.

## Author contributions

TW: corrected and proofreading of the manuscript, checking the statistics, literature research, submitting the manuscript, reviewing the statistics, prepared figures and tables, and wrote the manuscript. SB and SG: designed the concept, wrote the manuscript, and calculated the statistics. VP: wrote the manuscript, calculated the statistics, and prepared figures and tables. MV: reviewing the statistics, prepared figures and tables, and wrote the manuscript. LT: planning the study, writing the manuscript, reviewing the literature, managing the project, designed the concept, and calculated the statistics. All authors contributed to the article and approved the submitted version.

## Funding

This work was partly funded from the University of Osijek through the project IP2−2021 Low Resilience to Chronic Stress and Chronic Aging Diseases.

## Conflict of interest

The authors declare that the research was conducted in the absence of any commercial or financial relationships that could be construed as a potential conflict of interest.

## Publisher's note

All claims expressed in this article are solely those of the authors and do not necessarily represent those of their affiliated organizations, or those of the publisher, the editors and the reviewers. Any product that may be evaluated in this article, or claim that may be made by its manufacturer, is not guaranteed or endorsed by the publisher.
